# An evaluation of COVID-19 surveillance system in New Juaben South Municipality of Ghana: a cross-sectional study

**DOI:** 10.11604/pamj.2021.40.206.30715

**Published:** 2021-12-06

**Authors:** Hectoria Awekeya, Stephen Dajaan Dubik, Kingsley Amegah, Anthony Ashinyo, Francis Wuobar, Ekow Kaitoo, Winfred Ofosu, Mary Eyram Ashinyo

**Affiliations:** 1 Ghana College of Physicians and Surgeons, Faculty of Public Health, Accra, Ghana,; 2 Department of Quality Assurance, Eastern Regional Health Directorate, Ghana Health Service, Koforidua, Ghana,; 3 School of Allied Health Sciences, University of Development Studies, Tamale, Ghana,; 4 Department of Health Information, Hohoe Municipal Hospital, Hohoe, Ghana,; 5 National AIDS/STI Control Program, Accra, Ghana,; 6 Eastern Regional Hospital, Koforidua, Ghana,; 7 New Juaben South Municipality, Koforidua, Ghana,; 8 Office of the Regional Director of Health Service, Eastern Regional Health Directorate, Koforidua, Ghana,; 9 Department of Quality Assurance, Ghana Health Service Headquarters, Accra, Ghana

**Keywords:** COVID-19, pandemic, outbreak, Ghana, surveillance system evaluation

## Abstract

**Introduction:**

among others, the objectives of Ghana’s COVID-19 surveillance system are to rapidly detect, test, isolate and manage cases, to monitor trends in COVID-19 deaths and to guide the implementation and adjustment of targeted control measures. We therefore aimed to examine the operations of the COVID-19 surveillance system in New Juaben South Municipality, describe its attributes and explore whether its objectives were being met.

**Methods:**

we utilized a mixed method descriptive study design to evaluate the COVID-19 surveillance system in the New Juaben South Municipality of the Eastern Region of Ghana. Desk review and key informant interviews were carried out from 1^st^ February to 31^st^ March 2021 to measure nine surveillance system attributes as an approximation of its performance using the CDC’s 2013 updated surveillance system guidelines.

**Results:**

while the COVID-19 surveillance system in New Juaben South (NJS) was highly representative of its population, it was rated ‘moderate’ for its stability, flexibility, sensitivity and acceptability. The system was however characterized by a low performance on data quality, simplicity, timeliness and predictive value positive. The sensitivity and predictive value positive (PVP) of the system were 55.6% and 31.3% respectfully.

**Conclusion:**

while the surveillance system is only partially meeting its objectives, it is useful in the COVID-19 response in New Juaben South Municipality. System performance could improve with stigma reduction especially among health care workers, timely testing and simplification of surveillance forms and software.

## Introduction

The evaluation of surveillance systems is crucial for monitoring problems of public health importance and strengthening of health systems amid pandemics [1]. As at 2^nd^ July 2021, there were 182,319,261 confirmed cases including 3,954,324 deaths globally [2]. In Ghana, there were 96,067 confirmed cases including 796 deaths as at 28^th^ June 2021 [3]. Public health surveillance is the “ongoing systematic collection, analysis, interpretation and dissemination of data regarding health-related event for use in public health action to reduce morbidity and mortality and to improve health” [4,5]. Surveillance systems are instituted to study a variety of conditions in given populations. For the surveillance system to be effective, it must be developed around specific and well-defined objectives and the data collection done in a standardized manner, with the data analysis and dissemination carried out in a timely manner to the Ghana Health Service, Ministry of Health and partners [4,5].

Public health surveillance is one of the foundations for preventing and controlling the COVID-19 pandemic [6]. It is key to rapid case detection, which is crucial in containing and ending the COVID-19 pandemic worldwide [6]. Indeed, evidence suggest that COVID-19 transmission can be slowed or stopped through effective case detection, treatment, isolation and contact tracing [7]. In December 2019, the WHO initiated the COVID-19 surveillance system to detect the novel corona virus in countries across the world and also provided guidance to countries on operationalization of COVID-19 surveillance depending on transmission scenarios [8]. These surveillance systems could be community-based or facility-based. They have been implemented widely across Africa and other parts of the world even under very difficult situations [9-15].

Similarly in Ghana, COVID-19 surveillance activities were initiated in December 2019 [3]. Among others, the objectives of this surveillance system include the rapid detection, isolation, testing, and management of confirmed cases, the monitoring of trends in COVID-19 deaths, identification, follow-up and quarantine of contacts and the guidance of implementation and adjustment of targeted control measures. The surveillance system also seeks to evaluate the impact of the pandemic on health-care systems and society, monitor longer term epidemiologic trends and evolution of SARS-CoV-2 virus and contribute to the understanding of the co-circulation of SARS-CoV-2 virus and other pathogens [4]. The above objectives indicate the crucial role of Ghana´s surveillance system in containing the COVID-19 outbreak. However, there is little evidence about the operations of Ghana´s COVID-19 surveillance system since its inception in December 2019. The objective of this study was therefore to examine the operations of the COVID-19 surveillance system in New Juaben South (NJS) Municipality, describe its attributes and explore whether its objectives are being met.

## Methods

This study was a mixed method cross-sectional study using the updated Centres for Disease Control and Prevention (CDC) Guidelines on Surveillance System evaluation [4] to evaluate the COVID-19 surveillance system in NJS from 1^st^ February to 31^st^ March 2020. The NJS municipality is one of the 33 districts and municipalities of the Eastern Region of Ghana singularly accounting for 16.4% (674) ) of the Region's 4,177 COVID-19 confirmed cases, making it the district with the most cases in the region as at 30^th^April 2021 [16]. It is made up of eight sub municipalities with an estimated land size of 159 square kilometers representing approximately 0.6% of the total surface area of the Eastern Region [17]. With a growth rate of 2.6% [17], the population of the municipality was estimated at 217,389 in 2017 [18].

Desk reviews were conducted to review administrative reports and COVID-19 situational reports at national, eastern regional level as well as within the New Juaben South Municipality. Key informants were conveniently sampled based on their roles in the COVID-19 surveillance system and interviewed until saturation was attained. A semi structured questionnaire was administered to determine the operations, its attributes and usefulness of the surveillance system. The definitions of surveillance system attributes are shown in Table 1. In all, 16 key informants were interviewed including 2 disease control officers, 4 medical doctors, 3 physician assistants, 2 medical laboratory scientists, 2 community health nurses, 1 municipal director of health, 1 director of clinical services at the Eastern Regional Hospital, and 1 regional surveillance officer. Manual inductive and deductive thematic content analysis was then used to identify common themes across the qualitative data by generating codes and identifying common themes across the transcripts to answer our relevant research questions. For very complex and mystifying data, a thematic content analysis presents a flexible but systematic approach to data analysis and is suitable for this study. An inductive approach enabled the emergence of themes depending on what is contained in the data collected while a deductive approach enabled us to apply the surveillance evaluation tool as a framework to understand the complex actor interactions within the surveillance system.

**Table 1:** definitions, measurement and results of surveillance system attributes

Attribute	Definition	Measurement	Results
Simplicity	Degree of ease with reporting structure from one level to another for decision making and ease of operations	On a scale of low, moderate or high.	Low
Acceptability	The willingness of persons and organizations to participate in the surveillance system	On a scale of low, moderate or high.	Moderate
Flexibility	Extent to which system can adapt to changes in information need or reporting with little additional time, personnel or allocated funds.	On a scale of low, moderate or high.	Moderate
Stability	The stability of a surveillance system refers to its reliability and availability during.	On a scale of low, moderate or high.	Moderate
Data quality	Completeness and validity of the data recorded in the public health surveillance system.	On a scale of low, moderate or high.	Low
Sensitivity	At the level of case reporting, sensitivity refers to the proportion of cases detected by the surveillance system.	Number of suspected COVID-19 cases detected /Number of expected COVID-19 cases to be detected x 100	55.6
Predictive value positive (PVP)	The proportion of reported cases that actually have COVID-19.	PVP = Number of positive cases/Number of suspected cases reported x 100	31.3
Representativeness	Degree to which the system accurately describes the occurrence of COVID-19 over time and its distribution in the population by place and person.	On a scale of low, moderate or high.	High
Timeliness	Speed between steps in the COVID-19 surveillance system	On a scale of low, moderate or high.	Low

**Case definitions:** in Ghana, the following case definitions are used in all surveillance systems across the country. A suspected case of COVID-19 is any person presenting with fever (>38.0°C) or history of fever and symptoms of respiratory tract illness e.g. cough, difficulty in breathing and history of travel to, or residence in a location reporting person to person transmission of COVID-19 during the last 14 days prior to symptom onset. A probable case of COVID-19 is any suspected case for whom testing for the COVID-19 virus is inconclusive, or suspected case for whom testing could not be performed for any reason. A confirmed case of COVID-19 is a person with laboratory confirmation of COVID-19 infection irrespective of clinical signs and symptoms [19].

**Ethical considerations:** the study was carried out as part of an operational research to determine surveillance systems performance. Written informed consent was obtained from key informants. Administrative approval was obtained from the Eastern Regional Health Directorate Research Unit.

## Results

**Figure 1:**
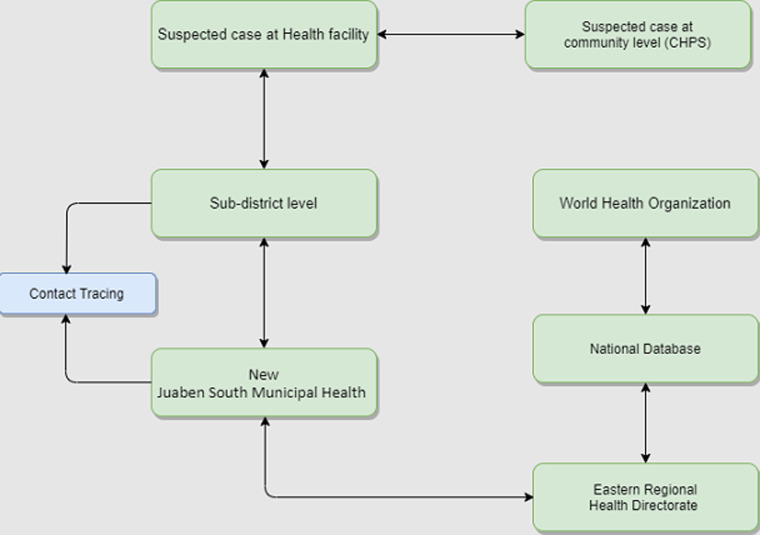
flow of COVID-19 surveillance information in New Juaben South

The reporting and dissemination of data and information is shown in Figure 1. Case detection happens both in healthcare facilities and community levels known as Community-based Health Planning and Services (CHPS). These cases are reported to sub-district levels who then report to the NJS Municipal Health Directorate (MHD). The MHD reports to the Eastern Regional Health Directorate (ERHD). This information is then reported to the national database of the Ghana Health Service which feeds into the surveillance system of the World Health Organization. The COVID-19 surveillance system in NJS municipal is mainly funded by Government of Ghana and other local and international organizations including development partners and private sector actors. We estimated the cost of surveillance per COVID-19 case to be $200.0 including cost of person-hours spent per case, cost of testing, transportation and cost of PPEs and logistics for sample collection.

We found that the COVID-19 surveillance system in this district was useful in containing and responding to the ongoing COVID-19 outbreak in the region, Ghana and the international COVID-19 surveillance space. Data collected through the surveillance system is used by stakeholders to monitor trends and magnitude of the disease as well as assess the effectiveness of preventive and control measures. Additionally, data generated through the surveillance system was used to make critical decisions. For instance, the system detected a worrying trend of HCWs infection leading to the introduction of a shift system in crowded facilities, the insistence of right use of PPEs through provision and monitoring on their use. The system was rated as moderately acceptable as healthcare workers who suspected cases did not always notify the authorities for testing and confirmation while some patients who had symptoms refused testing or isolation. We rated the data quality in the system 'low quality' as essential information on case base forms were either mostly blank (61%) or filled with 'non applicable' (72%). Data transfer in electronic forms from the district to the region and to the national level were also inconsistent. For instance, as at 31^st^ December, 2020, the municipal case count was 340, while 296 and 356 cases were reported for the municipality in the regional and national databases respectively for the same period. Simplicity of the surveillance system was perceived as low. Although various actors knew their roles in the surveillance system, levels of reporting were perceived as complex and multiple. The system was rated moderately flexible. Changes in reporting, case definitions, treatment guidelines and changes in policy direction or administrative arrangements were fairly accommodated without interruption in surveillance activities or need for additional time, personal and resources.

The NJS surveillance system was highly representative of the general population of new Juaben South municipality. Additionally, cases were reported from public, QUASI government and private facilities as well as from all levels of the health system namely community, sub-district, district and levels of care. Activities of the surveillance system were mostly not timely. While the average time a sample was taken following case detection was about two to three days, it took another two weeks minimum to receive test results at the peak of the pandemic. Due to this delay in transmitting results, contact tracing and actions to mitigate the spread of the disease were also delayed including moving of confirmed cases to treatment centres for appropriate management.

Operations of the surveillance system were considered to be moderately stable as there were no interruptions in times of difficulty such as erratic donor and government funding, supply of personal protective equipment (PPEs) and other logistics, breakdown of surveillance software and interrupted bar codes. The case reporting/detected sensitivity of the surveillance system estimates the proportion of cases detected by the surveillance system [4] and was calculated by dividing the number of suspected cases reported by the expected number of suspected cases to have been reported/detected (obtained from all reporting sites). The NJS surveillance system was rated as moderately sensitive with a value of 55.6% shown below:


$$Sensitivity=\frac{Number\;of\text{ suspected COVID-19 cases detected}}{Number\;of\text{ expected COVID-19 cases to be detected}}\times 100$$

Total number detected from reporting sites = 1,090. Expected number to be detected from reporting sites = 1959. Sensitivity = 1090/1959 x 100 = 55.6%. Predictive value positive (PPV) was defined as the proportion of reported cases that actually have the disease [5]. Data review of the records showed a low PVP of 31.1% as follows: Total COVID-19 suspected cases reported till 31^st^December 2020: 1,090; total COVID-19 positive cases detected till 31^st^December 2020: 342; PVP = number of positive cases/number of suspected cases reported x 100; PVP= 342/1090 x 100; PVP= 31.3%.

## Discussion

We aimed to examine the operations of the COVID-19 surveillance system in NJS municipality, describe its attributes and explore whether its objectives were being met. We found that the system was characterized by low simplicity and data quality but moderate stability, acceptability and flexibility. These suggest some difficulties in the structure and function of reporting, the use of the surveillance software and case base forms. Our findings are similar to those of Pakistan where systems stability, acceptability and flexibility were good [20] but differ from systems performance on other attributes in same study where the surveillance system was simple with good data quality [20]. The low simplicity in this study differ from the perspectives of healthcare workers in South Africa, where majority rated notifiable disease surveillance as simple [21].

The implications of poor data quality on systems performance include the possibility of wrong policies and decision making by managers at all levels as these decisions may not be addressing the actual gaps and needs of the COVID response in the district and region. Poor data quality of surveillance systems has been emphasized in a previous study in Kenya [22]. The surveillance system was rated as low performance on timeliness and PVP. This finding agrees with a similar that evaluated Ebola virus disease surveillance system in Sierra Leone where samples reached designated laboratory with 24-48 hours [23]. However, the PVP of 31.3% in this study is higher than that of Ilesanmi *et al*.in Tonkolili District of Sierra Leone [23]. Low timeliness in key activities of the system has implications on the ability to make a public health decision in a timely manner to control the spread and transmission of the virus in the municipality and beyond. The PVP of the system places emphasis on the proportion suspected cases that are confirmed by the surveillance system thus a low PVP may signify non-efficient use of resources as many false positives´ are being tested with system´s laboratory and other resources. These findings are similar to those of Pakistan where PVP was low [20] but relatively higher from findings in Ghana [24] where PVP for influenza-like illnesses was very low (7.4%). In Uganda, an evaluation of measles surveillance showed low PVP (8.6%) [25]. The low PVP found in this study may be an indication of low incidence of COVID-19 cases in NJS Municipality. The system was however useful, moderately acceptable and highly representative of the general population of NJS Municipal. The moderate flexibility and stability observed suggests its ability to accommodate changes in operations and its integration into and reliance on some resources of existing public health surveillance system. In their appraisal of the measles surveillance system in Ethiopia, Kalil and colleagues found that the surveillance system was useful, simple and flexible [26].

The surveillance system in NJS was rated as moderately sensitive at 55.6% of case detection. This suggests that the system is not able to detect almost half of the actual suspected cases. A surveillance system with low sensitivity could be threatened by the non-detection of new strains of COVID-19 for instance, which can increase their transmission and late detection within communities and healthcare facilities. The system could perform better if its challenges including persistent delay in diagnostic results, psychological support, low staff motivation, absence of an infectious disease centre, high level of stigma among the public and even more of concern among healthcare workers, inadequate PPEs, inadequate technical officers especially disease control officers, data discrepancies at different reporting levels and delays in reporting are addressed. Additionally, lengthy case base forms on surveillance software often led to fatigue of both the interviewee and the interviewer resulting in incomplete filling of these forms. The study did not capture the perspectives of community members on the COVID-19 surveillance system in the NJS municipality. Additionally, the study was limited to NJS Municipality. We recommend large scale studies to evaluate the COVID-19 surveillance system in Ghana.

## Conclusion

Although, the surveillance system for COVID-19 had palpable operational challenges, it has proven to be useful for decision making in the entire response system in the municipality and Ghana. The system partially meets its objectives as it is highly representative and moderately sensitive and acceptable to most stakeholders. However, timeliness, PVP and data quality were noted to be low. Key stakeholders have various health systems strengthening roles to play in order to mitigate the challenges identified to improve systems performance.

### What is known about this topic

- The COVID-19 pandemic has been devastating to the world and remains a public health threat to Ghana;
- Surveillance systems provide a means for the ongoing collection, analysis, interpretation and dissemination of health related data to provide information that can be used by key stakeholders to monitor and improve the health of populations.

### What this study adds

- COVID-19 surveillance systems may be challenged with stigma especially among healthcare workers, inadequate technical officers, data discrepancies, low staff motivation and a lack of an infectious disease centre;
- Enablers of COVID-19 surveillance systems performance include simple case base forms, less sophisticated surveillance software, adequate testing logistics and clear case definitions.
